# A Case of Urinary Infiltration of Scrotum, Mistaken for Orchitis

**Published:** 1896-04

**Authors:** Thomas H. Manley

**Affiliations:** New York


### A CASE OF URINARY INFILTRATION OF SCROTUM, MISTAKEN FOR ORCHITIS. PERINEAL URETHROTOMY, CONSECUTIVE GANGRENE AND DEATH.

BY THOMAS H. MANLEY, M.D.,
NEW YORK.

The following brief notes are submitted on a case which belongs to not an uncommon class; not that it presented any unusual or extraordinary features, but which sets forth the importance of the lesson which it conveys with respect to the necessity of accuracy in diagnosis in scrotal tumors or tumefactions; a subject but superficially considered in most text-books, and generally neglected even in many works specially devoted to genito-urinary surgery.

It might seem a simple matter to definitely recognize the pathological characters of scrotal enlargements by their symptomatology and a casual examination; but this is certainly an error, which all must concede who have had much experience in the surgical diseases of this region.

Maladies of a complicated character in the course of the spermatic-cord and in the scrotal pouch may involve, simultaneously or consecutively, so many different anatomical elements as to give rise to such a complexity of symptoms and so alter the relation of parts, that without a most painstaking examination, an error in diagnosis may be committed and, possibly in consequence, great misery entailed or even a life sacrificed.

It is only necessary to refer to some of the more ordinary lesions occupying those areas to understand how readily mistakes may be made.

For example, a bubo may be mistaken for a hernia, or *vice versa*. More than one capable surgeon has cut for hernia and had the chagrin, after laying down the law to a class of students, of exposing a lipoma, a cyst, or an abscess. There are many features of sarcoma, hygroma, and hydrocele, under various circumstances, so much alike, that none but a skilled expert can readily differentiate one from the other. Erysipelas, acute edema, and urinary infiltration of the scrotum present many features in common, which surely lead one into pitfalls, if he be unwary or unskilled.

It should be more generally known, that in many pathological states, and in neoplastic formations, a correct diagnosis, in this situation, may be impossible without a free exploratory incision into the tissues.

On the 11th of December, 1895, I was sent for to meet Dr. Deynard, of this city, in consultation, in the case of a man who had some form of scrotal disease.

I saw him at 2 P.M. He was forty-six years old; a driver by occupation, who had enjoyed good health and had never been ill in bed before within his recollection.

Five days previously he was seized with pain in the scrotum and sent for a physician. He was told, that he had inflammation in his testicles, and that they must be freely poulticed. Medicines to lull the pain were ordered, but the more he poulticed the greater was his distress; when in despair on the fifth day, the first doctor was discharged and a second called in.

Dr. Deynard at once recognized that his condition was serious, and insisted on a consultation with a surgeon.

When seen by me his condition was indeed desperate. He had the sunken, pinched features of one in the collapsing stage of fever. He had been having repeated chills, no sleep and constant thirst. The tongue was deeply furred, and there was the hay-like odor of septicemia distinctly evident; the pulse was 122 per minute, and temperature 103$\frac{4}{5}$. On oral examination I gathered that he had an old untreated stricture of the urethra, with difficulty in urination until the present trouble; when with each effort to void urine his agony was most intense and the swelling greater in the scrotum.

There was no history of syphilis, gonorrhea, or trauma, and hence, before the bedclothes were raised, my mind was made up, that there must have been a urethral stenosis with free discharge of urine into the peno-scrotal tissues and systemic poisoning from the resorption of the infected urinary elements. On exposure of the scrotum it was found to be swollen to the volume of an infant's head. It quite buried up the penis, had a boggy feel; and here and there, forward, near the peno-scrotal junction, anteriorly, there were scattered brownish, gray patches, suggestive of peripheral death or incipient gangrene. So great was the swelling that neither of the testes could be felt at all.

This was a grewsome case to take in hand. Neglect and ignorance had done their deadly work; but he had been a man of good physique who had never abused himself; besides, there were five helpless children dependent on his labors. The advice of Civiale to the surgeon, when in the presence of urinary-extravasation, whether pathological or traumatic, behind the triangular-ligament within the pelvis, or anterior in the perineo-scrotal region, was to operate "*sur le champ*"; but that time was past and now little could be done, except to perform a perineal urethrotomy through the structure and divert the urine outward. That operation I readily performed under cocaine-analgesia, with great relief following. But the following day nearly his whole scrotum fell away by phagedenic gangrene. The bare testes hung by the nude cords, only a small strip of the integuments remaining. Four days later a vast metastatic abscess developed in the left parotid-gland. The pus was deep-seated, requiring a very large incision to evacuate it. No improvement followed, and forty-eight hours later he sunk into coma and died.

Such was the melancholy ending of a life that should have been saved. How many more lives are thus annually lost through mistaken diagnosis, the records of which never see light in our medical chronicles?

But by mistakes we are later brought to avoid error, and if medical practitioners had only a little more moral courage and less zeal for self-laudation, and would let a little light in on the dark corners, as a sort of beacon to guide, particularly those about to set out on their voyage, how many would be warned of the breakers ahead, and what blessings they would bestow on poor suffering humanity? This case was the third which had come under my notice since April, 1895, of urethral trouble, resulting in impending or actual primary infiltration. The other two were traumatic; one was in hospital. This man, while intoxicated, had been kicked. The membranous section of the urethra had been suddenly driven up against the sharp edge of the descending ramus of the pubes and cut almost as neatly through as with a knife.

In this case, within twenty-four hours after the accident Wile's operation was performed by me, the urethra was immediately restored, and thus all danger of urinary infiltration was averted.

The other case was in an English mechanic who, while at work as a machinist, fell about five feet astride a narrow shaft striking heavily on the crotch. He continued work till evening, when trouble in urinating began. Medical aid was summoned, but it was insisted that his trouble was not traumatic but specific. The following morning he was brought to my office. At this time he showed signs of severe constitutional disturbance. Some blood was flowing from the meatus, and urination gave him great distress. Along the perineal raphe there was great distention of the spongy bodies of the urethra, with some discoloration of the overlying integument. After cleansing and cocainization of the urethra, a warm, well lubricated bougie was passed on to the bladder. After vesical irrigation, the catheter was left in until the second day. After its removal, it was evident that the linear or circular rent through the urethral mucous membrane had closed as there was no further trouble.

It is my intention, in the near future, to give in detail the history of these and several others, which have come under my care, of this interesting class, treated by various measures.

---

AT last the legislature of Ohio has passed a medical law establishing a State Board of Registration and Examination. The law is probably as good as could be gotten at this time, but the fact that the possession of a "diploma from a legally chartered medical institution in good standing" will constitute the right to practice under this law, and that the chief function of this board will consist in inspecting diplomas, will render the law less effective than our Ohio brethren would doubtless like. But even this much is a step in the right direction which we hope will be improved upon in time.

